# Inspiratory effort impacts the accuracy of pulse pressure variations for fluid responsiveness prediction in mechanically ventilated patients with spontaneous breathing activity: a prospective cohort study

**DOI:** 10.1186/s13613-023-01167-0

**Published:** 2023-08-17

**Authors:** Hui Chen, Meihao Liang, Yuanchao He, Jean-Louis Teboul, Qin Sun, Jianfen Xie, Yi Yang, Haibo Qiu, Ling Liu

**Affiliations:** 1 Jiangsu Provincial Key Laboratory of Critical Care Medicine, Department of Critical Care Medicine, Zhongda Hospital, School of Medicine, Southeast University, No. 87, Dingjiaqiao Road, Gulou District, Nanjing, 210009 People’s Republic of China; 2 Department of Critical Care Medicine, The First Affiliated Hospital of Soochow University, Soochow University, No. 899 Pinghai Road, Suzhou, 215000 People’s Republic of China; 3 Department of Critical Care Medicine, Changsha central hospital, University of South China, No. 161, South Shaoshan Road, Changsha, 410000 Hunan People’s Republic of China; 4 Department of Critical Care Medicine, Wuhan first hospital of Hubei Province, No 215 Zhongshan Avenue, Qiaokou District, Wuhan, 430000 People’s Republic of China; 5 Service de médecine intensive-réanimation, Hôpital de Bicêtre, Université Paris-Saclay, AP-HP, Inserm UMR S_999, Le Kremlin-Bicêtre, France

**Keywords:** Acute circulatory failure, Fluid responsiveness, Pulse pressure variation, Inspiratory effort

## Abstract

**Background:**

Pulse pressure variation (PPV) is unreliable in predicting fluid responsiveness (FR) in patients receiving mechanical ventilation with spontaneous breathing activity. Whether PPV can be valuable for predicting FR in patients with low inspiratory effort is unknown. We aimed to investigate whether PPV can be valuable in patients with low inspiratory effort.

**Methods:**

This prospective study was conducted in an intensive care unit at a university hospital and included acute circulatory failure patients receiving volume-controlled ventilation with spontaneous breathing activity. Hemodynamic measurements were collected before and after a fluid challenge. The degree of inspiratory effort was assessed using airway occlusion pressure (P_0.1_) and airway pressure swing during a whole breath occlusion (ΔP_occ_) before fluid challenge. Patients were classified as fluid responders if their cardiac output increased by ≥ 10%. Areas under receiver operating characteristic (AUROC) curves and gray zone approach were used to assess the predictive performance of PPV.

**Results:**

Among the 189 included patients, 53 (28.0%) were defined as responders. A PPV > 9.5% enabled to predict FR with an AUROC of 0.79 (0.67–0.83) in the whole population. The predictive performance of PPV differed significantly in groups stratified by the median value of P_0.1_ (P_0.1_ < 1.5 cmH_2_O and P_0.1_ ≥ 1.5 cmH_2_O), but not in groups stratified by the median value of ΔP_occ_ (ΔP_occ_ < − 9.8 cmH_2_O and ΔP_occ_ ≥ − 9.8 cmH_2_O). Specifically, in patients with P_0.1_ < 1.5 cmH_2_O, PPV was associated with an AUROC of 0.90 (0.82–0.99) compared with 0.68 (0.57–0.79) otherwise (p = 0.0016). The cut-off values of PPV were 10.5% and 9.5%, respectively. Besides, patients with P_0.1_ < 1.5 cmH_2_O had a narrow gray zone (10.5–11.5%) compared to patients with P_0.1_ ≥ 1.5 cmH_2_O (8.5–16.5%).

**Conclusions:**

PPV is reliable in predicting FR in patients who received controlled ventilation with low spontaneous effort, defined as P_0.1_ < 1.5 cmH_2_O.

*Trial registration* NCT04802668. Registered 6 February 2021, https://clinicaltrials.gov/ct2/show/record/NCT04802668

**Supplementary Information:**

The online version contains supplementary material available at 10.1186/s13613-023-01167-0.

## Introduction

Fluid administration is an integral intervention in the management of patients with acute circulatory failure [1, 2]. Fluid therapy can reverse a hypovolemic state and improve tissue oxygenation, while excessive fluid loading is associated with increased mortality [3]. Nevertheless, only half the critically ill patients could benefit from fluid administration in terms of increased cardiac output (CO) [4]. Hence, it is essential to assess fluid responsiveness (FR) to achieve appropriate fluid management in circulatory failure patients.

Pulse pressure variation (PPV) is a valuable index to predict fluid responsiveness in patients receiving mechanical ventilation with a tidal volume (*V*_T_) of at least 8 mL/kg and is not valid in patients with spontaneous breathing activity [5, 6]. However, persistent spontaneous breathing activity during mechanical ventilation is common in real clinical practice, and it is currently recommended to allow patients to use respiratory muscles partially [7, 8]. Assessment of FR is a difficult challenge in such a situation. Previous studies indicated that the predictive performance of PPV in patients with spontaneous breathing was variable and ranged from 0.68 to 0.98 [9]. None of these studies took into account the influence of the strength of inspiratory effort. Variable inspiratory efforts are associated with variable changes in intrathoracic pressure and thus lead to both false-positive or false-negative PPV values [10]. During the controlled ventilation, the low inspiratory effort could trigger the ventilator without substantially affecting the change of intrathoracic pressure. Hence, we hypothesized that PPV might be valid for the prediction of FR in patients with controlled ventilation and low inspiratory effort.

Therefore, we conducted a prospective study to assess the performance of PPV for the prediction of FR in acute circulatory failure patients who received controlled ventilation, but with spontaneous breathing activity. We also aimed to explore whether inspiratory effort impacts the predictive performance of PPV and to prove whether PPV can be valuable in patients with low inspiratory effort.

## Methods

### Setting and patients

This prospective study was conducted in the intensive care unit (ICU) of Zhongda Hospital, Southeast University from March 2021 to March 2022. Adult patients who fulfilled the definition of acute circulatory failure were eligible for inclusion. Acute circulatory failure was defined as the presence of systolic blood pressure (SBP) ≤ 90 or a > 40 mmHg decline of systolic arterial pressure in patients known to be hypertensive or mean arterial pressure (MAP) ≤ 70 mmHg or requiring vasopressors to maintain SBP > 90 mmHg or MAP > 70 mmHg, along with signs of hypoperfusion (urinary flow < 0.5 ml/kg/min for > 2 h, or presence of skin mottling or blood lactate concentration ≥ 2.0 mmol/L) [11]. All included patients were ventilated with a controlled-volume mode but with spontaneous effort. Patients having cardiac arrhythmias, valvular heart disease, right ventricular dysfunction, intracardiac shunt, air leakage through chest drains, intra-abdominal hypertension, and pregnancy or urgently requiring a fluid bolus were excluded.

This study was approved by the Zhongda Hospital Ethics Committee (Southeast University, Nanjing, China, approval ID: 2020ZDSYLL274-P01). Written informed consent was obtained from each patient or their legal representative prior to enrollment in this study. Our study was registered in ClinicalTrials.gov (NCT04802668, the current study was part of the registered trial).

### Study design

**Fig. 1:**
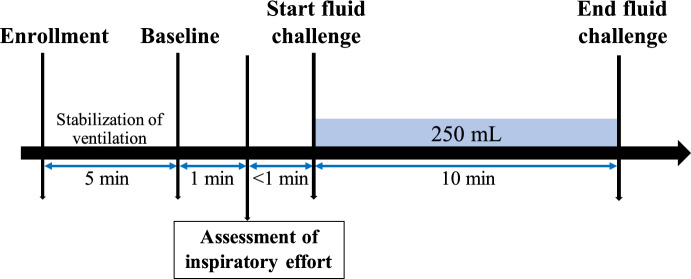
Study design

At enrollment in this study, all included patients were sedated and ventilated using the volume-controlled mode (Servo-I, Maquet, Solna, Sweden). The V_T_ was adjusted to 6–8 mL/kg predicted body weight (PBW), and other parameters were set according to the decision of the clinicians in charge. Patients also had a central venous catheter and a thermistor-tipped arterial catheter in the femoral artery connected to a transpulmonary thermodilution device (PiCCO, Philips Medizin System, Boeblingen, Germany). After a 5-min stabilization of ventilation (Baseline), the inspiratory effort was assessed by airway occlusion pressure (P_0.1_) and end-expiratory occlusion. Then, a fluid challenge was performed with a 250 ml saline bolus infused within 10 min (Fig. 1). Patients were classified as fluid responders if an increase in CO greater than or equal to 10% followed fluid administration [12].

All patients fulfilled the diagnosis of acute circulatory failure (see above). We assessed FR in each patient to decide the fluid management strategy. During the study period, there was no modification in the doses of vasopressor or sedative agents and no other fluid infusion. This study was stopped in cases of (1) new cardiac arrhythmias, (2) a > 20 mmHg decline of MAP from baseline, or (3) oxygen saturation (SpO_2_) < 90% for > 2 min.

### Measurements

At baseline, respiratory parameters were obtained including FiO_2_, V_T_, respiratory rate (set and observed) and positive end-expiratory pressure (PEEP). The P_0.1_, which is the drop in airway pressure (P_aw_) 100 ms after the onset of inspiration during an end-expiratory airway occlusion, was directly recorded from the ventilator by activating the P_0.1_ maneuver [13]. Three consecutive P_0.1_ measurements were averaged. End-expiratory occlusion was then performed and maintained for the duration of a single breath (confirmed by the return of P_aw_ to baseline). The maximal deflection in P_aw_ from PEEP during each occlusion was recorded as a measurement of occlusion pressure (ΔP_occ_) [14].

The vasopressor dose at baseline was calculated using the norepinephrine equivalent (NEE) dose. The NEE (µg/kg/min) was calculated as [norepinephrine (µg/kg/min) + epinephrine (µg/kg/min) + dopamine (µg/kg/min)/150 + vasopressin (U/min)/0.4 + phenylephrine (µg/kg/min)/10] [15]. Arterial blood gas analysis was also performed at baseline. Hemodynamic measurements collected before and after fluid challenge included heart rate (HR), MAP, central venous pressure (CVP), PPV, and CO. Three consecutive measurements of PPV were averaged. The CO was obtained by the average of three transpulmonary thermodilution measurements using 15 ml cold saline with the PiCCO system.

### Statistical analysis

Based on previous studies, we assumed that PPV predicted FR with an AUROC of 0.90 in patients with low inspiratory effort and with an AUROC of 0.75 in patients with high inspiratory effort [16, 17]. A sample size of 85 patients from each group achieves 85% power at a 2-sided alpha of 5% to detect a difference of 0.15 between groups (PASS V.11).

Values are presented as the mean (standard deviation) or median [interquartile range (IQR)] for continuous variables as appropriate and as the total number (percentage) for categorical variables. Comparisons between groups (according to the presence of FR and according to the median values of P_0.1_ and of ΔP_occ_) were made using the *X*^2^ test or Fisher’s exact test for categorical variables and Student’s *t*-test or Mann–Whitney *U* test for continuous variables as appropriate. Hemodynamic variables before and after fluid challenge were compared using paired *t*-tests or the Wilcoxon signed-rank test after normality test.

We first employed receiver operating characteristic (ROC) curves to assess the capacity of PPV to predict FR. The ROC data were presented as the areas under the ROC curve (AUROC) value (with a 95% confidence interval), sensitivity (with a 95% confidence interval), and specificity (with a 95% confidence interval). The optimal cut-off value of PPV was determined by the Youden Index (sensitivity + specificity -1). Additionally, we also use a two-step gray zone approach to evaluate the predictive ability of PPV, which was reported elsewhere [10]. The gray zone indicated two cut-offs between which the diagnosis of FR remained uncertain, and was defined as the values presenting with either sensitivity less than 90% or specificity less than 90% [18].

To explore the impact of inspiratory effort on the capacity of PPV to predict FR, we divided patients into two groups with different degrees of inspiratory effort, based on the median value of P_0.1_ (P_0.1_ < 1.5 cmH_2_O and P_0.1_ ≥ 1.5 cmH_2_O) and ΔP_occ_ (ΔP_occ_ ≥ − 9.8 cmH_2_O and ΔP_occ_ < − 9.8 cmH_2_O). We first constructed univariable logistical regression models to identify the association between degrees of inspiratory effort and correct classification (true-positive and true-negative results) of FR status at a PPV cut-off value of 9.5% (obtained from the first step) [19]. We then compared the PPV performance, including AUROCs and the gray zone between groups, and the AUROCs were compared using the Hanley-McNeil test [20]. Considering that patients with low inspiratory efforts were ventilated with a higher tidal volume than patients with high inspiratory efforts, we also compared AUROCs between groups with different inspiratory efforts after adjusting PPV for tidal volume using bootstrap. All statistical analyses were performed using R (version 4.0.3), and *p* < 0.05 was considered statistically significant.

## Results

### Patient characteristics

A total of 189 patients were included in the final analysis (Additional file 1: Fig S1). Their mean age was 66.3 (13.3) yrs. The sequential organ failure assessment (SOFA) score at enrollment was 9.9 (3.5). Septic shock was the most frequent type of circulatory failure, and the proportion was as high as 88%. The patients received vasopressor at a median dose of 0.33 (IQR: 0.15–0.57) µg/kg/min NEE. 91.3% patients received norepinephrine, 12.5% patients received epinephrine, and 6.7% patients received vasopressin. At inclusion, patients were ventilated with a tidal volume of 7.0 (1.0) mL/kg PBW, a PEEP of 5.1 (0.5) cmH_2_O, and a respiratory rate of 19.4 (5.3) breaths/min. In the whole population, P_0.1_ was 1.5 (IQR: 0.8–2.8) cmH_2_O, and ΔP_occ_ was − 9.8 [IQR: − 14.0 to − 3.7] cmH_2_O.

Fifty-three patients (28%) were defined as fluid responders. Comparisons between responders and non-responders are shown in Additional file 1: Table S1. Most baseline characteristics showed no significant differences between the two groups. Changes in hemodynamic parameters are shown in Additional file 1: Table S2. The changes in the MAP and CO after volume expansion were significantly larger in the responders than in the non-responders.

### Predictive performance of PPV in the whole population

**Fig. 2:**
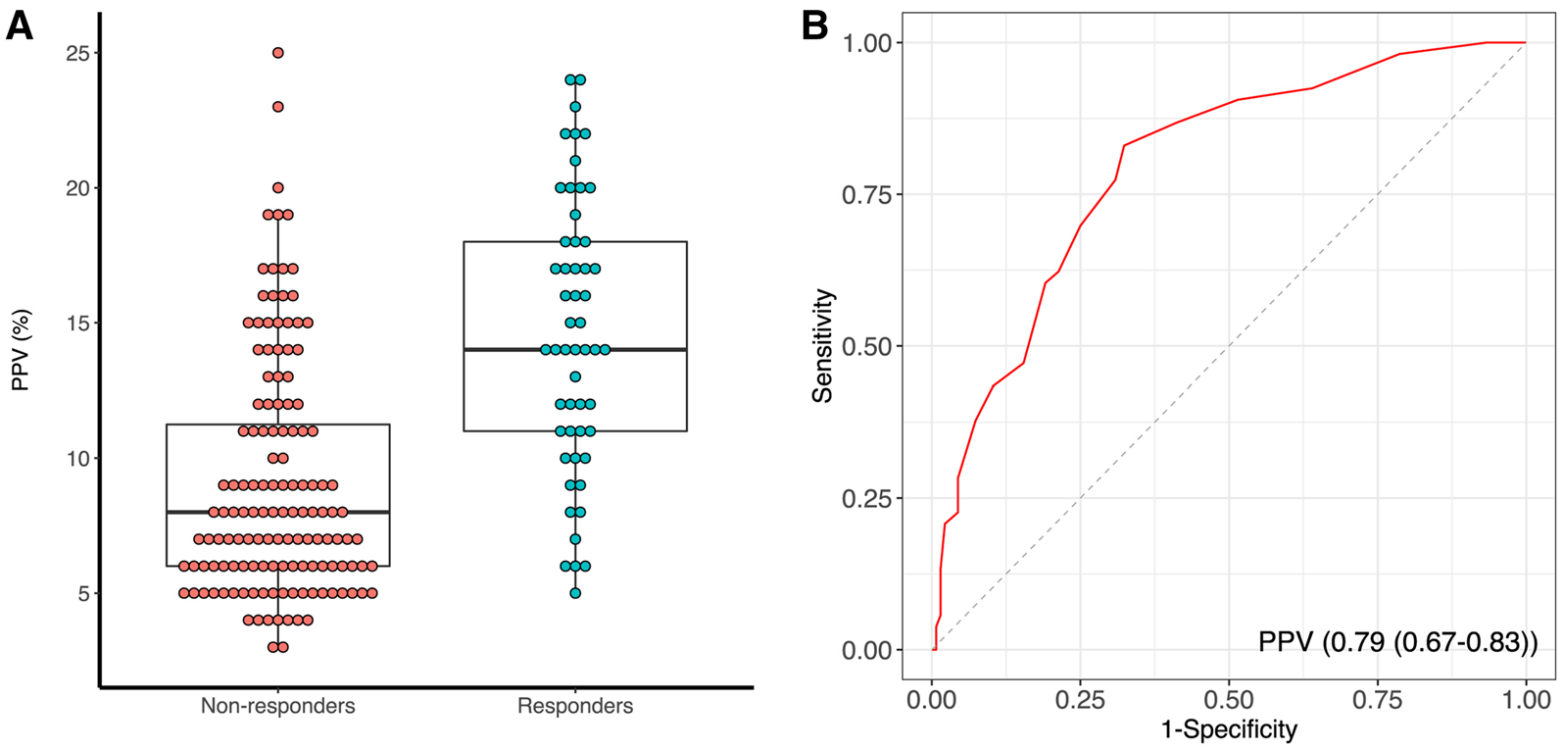
Predictive performance of pulse pressure variation to predict fluid responsiveness in whole acute circulatory failure patients. **A**: Comparison of pulse pressure variation between responders and non-responders; **B**: Receiver operating characteristic curves for pulse pressure variation to detect fluid responsiveness. *PPV* pulse pressure variation

Baseline PPV was significantly higher in responders compared to non-responders. A PPV > 9.5% enabled to predict FR with an AUROC of 0.79 (0.67–0.83), and sensitivity and specificity were 83% (66–92%) and 69% (58–82) %, respectively (Fig. 2). The positive predictive value was 51.1 (43.4–61.4) %, and negative predictive value was 91.2% (85.1–96.2) %. The Youden index was 0.51. The gray zone was 8.5–15.5% (33% of the included patients) (Additional file 1: Fig S2).

### Comparisons of PPV performance stratified by the median value of P_***0.1***_

Compared to patients with P_0.1_ ≥ 1.5 cmH_2_O, patients with P_0.1_ < 1.5 cmH_2_O had a significantly higher ΔP_occ_ (− 3.6 [− 7.4 to − 2.0] cmH_2_O vs. − 13.4 [− 22.1 to − 8.9] cmH_2_O, *p* < 0.001), a higher tidal volume (7.2 (1.0) ml/kg PBW vs. 6.8 (1.0) ml/kg PBW, *p* = 0.002), and a lower total respiratory rate (17.7 (3.3) bpm vs. 20.9 (6.2) bpm, *p* < 0.001). Other parameters between the two groups were not significantly different. Comparisons of clinical characteristics between groups are shown in Table 1. The changes in CO after volume expansion were substantially larger in the responders than in the non-responders in both two groups (Table 2).

**Table 1:** Baseline characteristics of included patients at enrollment stratified by the median value of P_0.1_ and ΔP_occ_

	Stratified by P_0.1_			Stratified by ΔP_occ_		
	P_0.1_ < 1.5 cmH_2_O	P_0.1_ ≥ 1.5 cmH_2_O	*P* value	ΔP_occ_ < − 9.8 cmH_2_O	ΔP_occ_ ≥ − 9.8 cmH_2_O	*P* value
Number	90	99	–	93	96	–
Age, year	66.8 (12.8)	66.0 (13.9)	0.69	67.5 (13.9)	65.2 (12.7)	0.23
Gender, male (%)	51 (56.7)	70 (70.7)	0.063	66 (71.0)	55 (57.3)	0.071
BMI, kg/m^2^	23.8 (3.6)	23.7 (4.1)	0.79	24.1 (4.3)	23.5 (3.3)	0.31
APACHE II	25.0 (6.3)	24.0 (6.3)	0.26	23.4 (6.0)	25.5 (6.4)	0.020
SOFA score	9.7 (3.3)	10.0 (3.7)	0.58	9.8 (3.4)	9.9 (3.6)	0.75
Acute circulatory failure origin, *n* (%)						
Septic shock	78 (86.7)	89 (89.9)	0.64	83 (89.2)	84 (87.5)	0.88
Cardiogenic shock	1 (1.1)	3 (3.0)	0.68	4 (4.3)	0 (0.0)	0.12
Neurogenic shock	6 (6.7)	6 (6.1)	1.0	2 (2.2)	10 (10.4)	0.042
Hypovolemic shock	5 (5.6)	5 (5.1)	1.0	5 (5.4)	5 (5.2)	1.0
Respiratory parameters at enrollment						
FiO_2_	0.50 (0.18)	0.48 (0.17)	0.31	0.47 (0.16)	0.50 (0.19)	0.27
Tidal volume, ml	429.8 (96.4)	420.7 (87.5)	0.50	423.52 (80.68)	426.50 (101.70)	0.82
Tidal volume/PBW, ml/kg	7.2 (1.0)	6.8 (1.0)	0.002	6.9 (1.0)	7.1 (1.00)	0.041
Respiratory rate (Set), bpm	15.0 (2.3)	15.4 (2.6)	0.25	15.3 (2.4)	15.1 (2.5)	0.54
Respiratory rate (Observed), bpm	17.7 (3.3)	20.9 (6.2)	< 0.001	20.9 (6.1)	17.9 (3.8)	< 0.001
Peak pressure, cmH_2_O	23.8 (5.4)	25.2 (7.1)	0.21	24.8 (5.7)	24.1 (6.6)	0.53
Plateau pressure, cmH_2_O	17.5 (4.0)	18.0 (5.3)	0.054	18.2 (5.2)	17.3 (4.2)	0.20
Driving pressure, cmH_2_O	11.6 (4.1)	11.9 (5.0)	0.72	12.1 (4.9)	11.5 (4.2)	0.38
Compliance, ml/cmH_2_O	38.7 [30.0, 50.7]	35.3 [27.9, 47.1]	0.46	33.6 [27.9, 46.3]	39.0 [31.3, 51.8]	0.13
PEEP, cmH_2_O	5.0 (0.0)	5.1 (0.7)	0.24	5.1 (0.7)	5.0 (0.0)	0.21
P_0.1_, cmH_2_O	0.7 [0.2, 1.0]	2.8 [1.9, 5.0]	< 0.001	2.3 [1.5, 5.0]	0.8 [0.3, 1.6]	< 0.001
ΔP_occ_, cmH_2_O	− 3.6 [− 7.4, − 2.0]	− 13.4 [− 22.1, − 8.9]	< 0.001	− 14.6 [− 23.1, − 10.7]	-3.7 [-5.9, -2.0]	< 0.001
PaO_2_/FiO_2_, mmHg	223 [164, 287]	223 [177, 287]	0.88	225 [201, 290]	220 [159, 268]	0.19
Hemodynamic parameters at enrollment						
Heart rate, beats/min	98.2 (21.7)	98.5 (19.4)	0.91	96.1 (18.2)	90.0 (15.2)	0.013
HR/RR	5.15 (1.06)	4.90 (1.37)	0.16	4.85 (1.27)	5.18 (1.17)	0.065
Vasopressor dose, μg/kg/min NEE	0.32 [0.15, 0.50]	0.37 [0.16, 0.57]	0.20	0.37 [0.15, 0.57]	0.31 [0.16, 0.58]	0.43
Lactate, mmol/L	3.4 (2.7)	3.5 (2.6)	0.83	3.2 (2.6)	3.6 (2.7)	0.28
Fluid responders	22 (24.4)	31 (31.3)	0.37	32 (34.4)	21 (21.9)	0.079

*BMI* Body mass index, *APACHE II* Acute physiology and chronic health score II, *SOFA* Sequential organ failure assessment, *PBW* Predicted body weight, *PEEP* Positive end-expiratory pressure, *P*_*0.1*_ Airway occlusion pressure, *ΔP*_*occ*_ Airway pressure swing during a whole breath occlusion, *PaO*_*2*_*/FiO*_*2*_ Arterial partial pressure of oxygen/fraction of inspired oxygen, *HR/RR* Heart Rate/Respiratory Rate (Observed), *NEE* Norepinephrine equivalent

**Table 2:** Effects of volume expansion on hemodynamic parameters in fluid responders and non-responders stratified by the median value of P_0.1_

	P_0.1_ < 1.5 cmH_2_O				P_0.1_ ≥ 1.5 cmH_2_O			
	responders (*n* = 22)		non-responders (*n* = 68)		responders (*n* = 31)		non-responders (*n* = 68)	
	Baseline	After fluids	Baseline	After fluids	Baseline	After fluids	Baseline	After fluids
MAP, mmHg	77.6 (11.0)	83.2 (15.0)	89.0 (13.7)^$^	91.3 (14.1)^&^	79.4 (13.5)	84.5 (12.5)	88.9 (13.5)^$^	91.0 (13.2)^&^
CVP, mmHg	8.1 (3.5)	7.7 (4.1)	8.9 (3.3)	10.6 (4.5)^&#^	8.1 (4.3)	8.8 (4.6)	10.2 (4.3)^$^	11.3 (4.6)^&^
PPV, %	15.5 (5.4)	13.3 (6.5)	7.1 (3.8)^$^	6.4 (2.3)^&^	11.9 (6.3)	11.6 (4.5)	8.0 (3.9)^$^	8.8 (5.0)^&^
SVV, %	15.6 (7.4)	12.2 (6.1)	6.4 (2.3)^$^	6.2 (3.5)^&^	11.9 (7.0)	11.3 (5.0)	8.5 (5.3)^$^	8.4 (4.8)^&^
Cardiac output, L/min	4.9 (1.0)	6.1 (1.4)^*^	6.3 (1.5)^$^	6.4 (1.6)	5.8 (1.3)	7.2 (2.1)^*^	6.93 (1.96)^$^	7.0 (2.21)
Cardiac index, L/min/m^2^	2.8 (0.5)	3.5 (0.8)^*^	3.8 (0.9)^$^	3.8 (0.9)	3.3 (0.7)	4.2 (1.0)^*^	3.96 (0.97)^$^	4.0 (1.0)

^*^p < 0.05: After Fluids versus Baseline (responders)

^#^*p* < 0.05: After Fluids versus Baseline (non-responders)

^$^*p* < 0.05: non-responders versus responders (Baseline)

^&^*p* < 0.05: non-responders versus responders (After Fluids)

*MAP* Mean arterial pressure, *CVP* Central venous pressure, *PPV* Pulse pressure variation, *SVV* Stroke volume variation

**Fig. 3:**
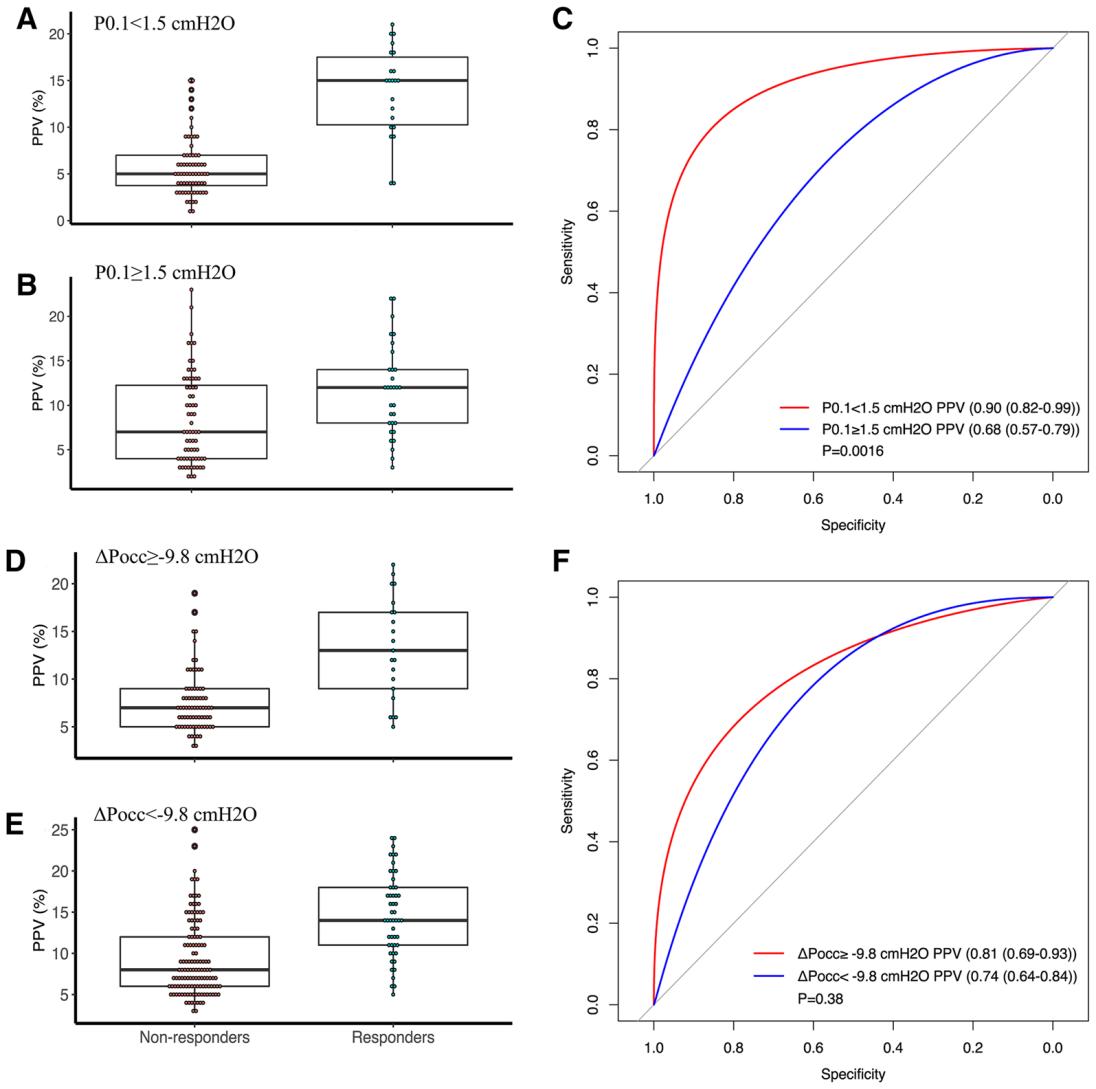
Accuracy of pulse pressure variation to predict fluid responsiveness in subgroups of patients stratified by the different degrees of inspiratory effort. **A**: Comparison of pulse pressure variations between responders and non-responders in patients with P_0.1_ ≥ 1.5 cmH_2_O; **B**: Comparison of pulse pressure variation between responders and non-responders in patients with P_0.1_ < 1.5 cmH_2_O; **C**: Comparison of pulse pressure variation performance between patients with P_0.1_ < 1.5 cmH_2_O and patients with P_0.1_ ≥ 1.5 cmH_2_O; **D**: Comparison of pulse pressure variation between responders and non-responders in patients with ΔP_occ_ ≥ − 9.8 cmH_2_O; **E**: Comparison of pulse pressure variation between responders and non-responders in patients with ΔP_occ_ < − 9.8 cmH_2_O; **F**: Comparison of pulse pressure variation performance between patients with ΔP_occ_ ≥ − 9.8 cmH_2_O and patients with ΔP_occ_ < − 9.8 cmH_2_O. *P*_*0.1*_ Airway occlusion pressure, *PPV* pulse pressure variation, *ΔP*_*occ*_ Airway pressure swing during a whole breath occlusion

**Fig. 4:**
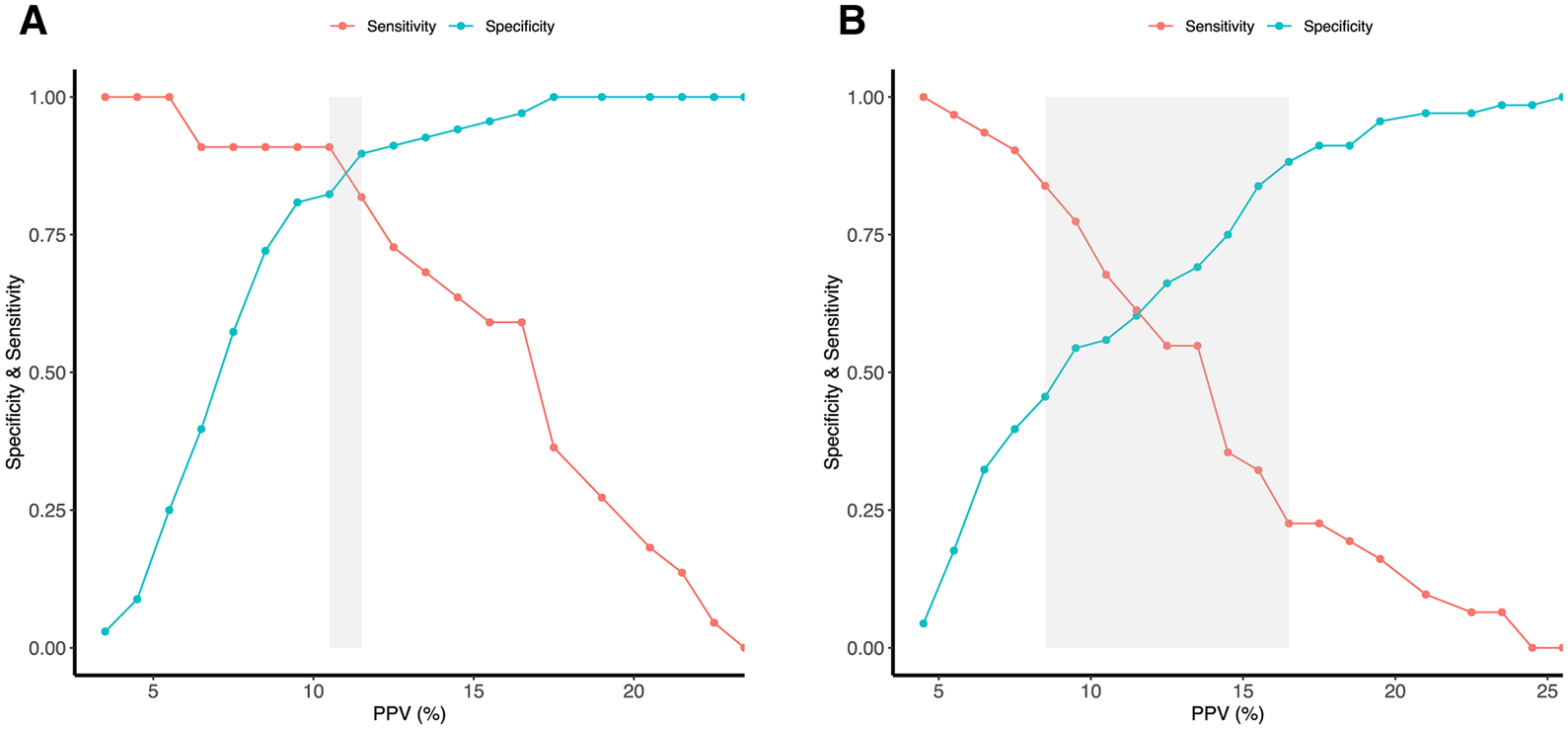
Gray zone of pulse pressure variation to predict fluid responsiveness patients with P_0.1_ < 1.5 cmH_2_0 (10.5–11.5%) (**A**) and P_0.1_ ≥ 1.5 cmH_2_0 (8.5–16.5%) (**B**). *P*_*0.1*_ Airway occlusion pressure, *PPV* pulse pressure variation

The proportion of FR was 24% in patients with low P_0.1_ and 31% in patients with high P_0.1_. Patients with P_0.1_ ≥ 1.5 cmH_2_O were associated with an increased probability of incorrect classification of FR using PPV (Additional file 1: Table S3). Additionally, in patients with P_0.1_ < 1.5 cmH_2_O, PPV predicted FR with an AUROC of 0.90 (0.82–0.99), which was significantly higher compared to 0.68 (0.57–0.79) in patients with P_0.1_ ≥ 1.5 cmH_2_O (*p* = 0.0016). The Youden indexes were 0.73 and 0.32, respectively. The cut-off values were 10.5% and 9.5%, respectively (Table 4 and Fig. 3). Besides, patients with P_0.1_ < 1.5 cmH_2_O had a narrow gray zone (10.5–11.5%) that only included 2/90 patients, while patients with P_0.1_ ≥ 1.5 cmH_2_O had a broad gray zone (8.5–16.5%) that included 48/99 patients (Fig. 4). After adjusting for tidal volume, the adjusted AUROC was 0.91 (0.83–0.99) in patients with P_0.1_ < 1.5 cmH_2_O compared to 0.67 (0.55–0.78) in patients with P_0.1_ ≥ 1.5 cmH_2_O, and the difference was also significantly (*p* < 0.001).

### Comparisons of PPV performance stratified by the median value of ΔP_occ_

**Fig. 5:**
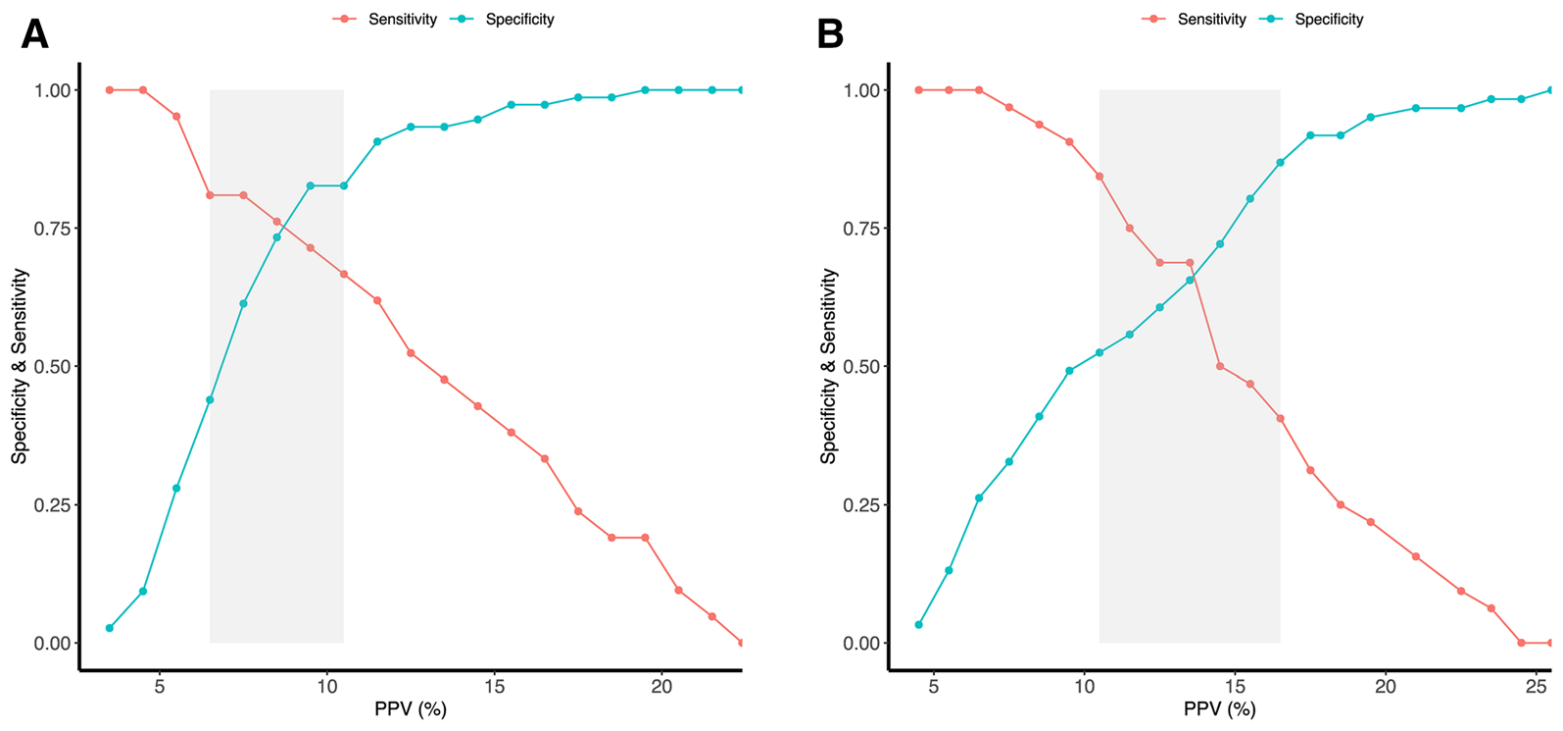
Gray zone of pulse pressure variations to predict fluid responsiveness patients with ΔP_occ_ ≥ − 9.8 cmH_2_O (6.5–10.5%) (**A**) and ΔP_occ_ < − 9.8 cmH_2_O (10.5–16.5%) (**B**). *PPV* pulse pressure variation, *ΔP*_*occ*_ Airway pressure swing during a whole breath occlusion

The differences between groups stratified by ΔP_occ_ were similar to groups stratified by P_0.1_ (Table 1). The changes in CO after volume expansion were more significant in the responders than in the non-responders in both groups (Table 3). The proportion of FR was 34% in patients with ΔP_occ_ < − 9.8 cmH_2_O, and 22% in patients with ΔP_occ_ ≥ − 9.8 cmH_2_O. Patients with ΔP_occ_ < − 9.8 cmH_2_O were associated with an increased probability of incorrect classification of FR using PPV (Additional file 1: Table S3). PPV predicted FR with an AUROC of 0.81 (0.69–0.93) in patients with ΔP_occ_ ≥ − 9.8 cmH_2_O, which was higher than 0.74 (0.64–0.84) in patients with ΔP_occ_ < − 9.8 cmH_2_O, while the difference did not differ significantly (*p* = 0.38) (Table 4 and Fig. 3). The Youden indexes were 0.54 and 0.40, respectively. The cut-off values were 10.0% and 9.5%, respectively. Additionally, patients with ΔP_occ_ ≥ − 9.8 cmH_2_O exhibited a gray zone (6.5–10.5%) that included 32/96 patients, compared to patients with ΔP_occ_ < − 9.8 cmH_2_O that had a gray zone (10.5–16.5%) included 35/93 patients (Fig. 5). The adjusted AUROC was 0.80 (0.66–0.93) for patients with ΔP_occ_ ≥ − 9.8cmH_2_O compared to 0.74 (0.64–0.84) for patients with ΔP_occ_ < − 9.8cmH_2_O, while the difference did not differ significantly (*p* = 0.49).

**Table 3:** Effects of volume expansion on hemodynamic parameters in fluid responders and non-responders stratified by the median value of ΔP_occ_

	ΔP_occ_ < − 9.8 cmH_2_O				ΔP_occ_ ≥ − 9.8 cmH_2_O			
	responders (*n* = 32)		non-responders (*n* = 61)		responders (*n* = 21)		non-responders (*n* = 75)	
	Baseline	After fluids	Baseline	After fluids	Baseline	After fluids	Baseline	After fluids
MAP, mmHg	80.0 (12.3)	84.6 (13.0)	86.4 (11.8)^$^	88.2 (12.6)	76.7 (12.6)	83.0 (14.40)	91.0 (14.6)^$^	93.5 (14.1)^&^
CVP, mmHg	8.0 (3.5)	8.7 (4.2)	10.1 (3.8)^$^	11.3 (4.6)^&^	8.3 (4.7)	7.9 (4.7)	9.2 (3.9)	10.7 (4.5)^&#^
PPV, %	13.7 (6.6)	12.0 (4.6)	7.6 (3.5)^$^	8.4 (4.7)^&^	12.6 (5.5)	12.7 (6.6)	7.4 (4.1)^$^	6.9 (3.2)^&^
SVV, %	14.1 (7.8)	11.4 (5.6)	8.3 (4.6)^$^	8.3 (4.6)^&^	12.0 (6.4)	11.9 (5.2)	6.7 (5.1)^$^	6.5 (3.9)^&^
Cardiac output, L/min	5.6 (1.3)	7.1 (2.1)^*^	6.9 (1.8)^$^	7.0 (1.9)	5.2 (1.2)	6.3 (1.6)^*^	6.4 (1.7)^$^	6.5 (1.8)
Cardiac index, L/min/m^2^	3.1 (0.70)	4.0 (1.0)^*^	4.0 (0.9)^$^	4.0 (1.0)	3.1 (0.7)	3.77 (0.90)^*^	3.8 (0.9)^$^	3.8 (0.9)

^*^p < 0.05: After Fluids versus Baseline (responders);

^#^*p* < 0.05: After Fluids versus Baseline (non-responders);

^$^*p* < 0.05: non-responders versus responders (Baseline)

^&^*p* < 0.05: non-responders versus responders (After Fluids)

*MAP* Mean arterial pressure, *CVP* Central venous pressure, *PPV* Pulse pressure variation, *SVV* Stroke volume variation

**Table 4:** The accuracy of pulse pressure variations to predict fluid responsiveness in patients with different degrees of inspiratory effort

	AUROC	Threshold, %	Sensitivity, %	Specificity, %	Positive predictive value, %	Negative predictive value, %
P_0.1_						
< 1.5 cmH_2_O	0.90 (0.82–0.99)^*^	> 10.5	86.8 (76.5–97.1)	90.9 (72.7–100)	68.0 (55.0–89.5)	96.6 (91.2–100)
≥ 1.5 cmH_2_O	0.68 (0.57–0.79)	> 9.5	83.9 (45.2–100)	51.5 (30.9–86.8)	44.3 (37.8–59.5)	87.9 (76.7–100)
ΔP_occ_						
< − 9.8 cmH_2_O	0.74 (0.64–0.84)	> 9.5	90.6 (46.9–100)	54.1 (36.1–91.8)	50.9 (43.3–76.7)	90 (76.4–100)
≥ − 9.8 cmH_2_O	0.81 (0.69–0.93)^#^	> 10	71.4 (47.6–90.5)	87.3 (69.0–97.2)	60.9 (41.9–84.6)	91.2 (85.3–96.8)

^*^p = 0.0016 versus P_0.1_ ≥ 1.5 cmH_2_0;

^#^*p* = 0.38 versus ΔP_occ_ < − 9.8 cmH_2_O

*AUROC* Area under the receiver operating characteristic curve, *P*_*0.1*_ Airway occlusion pressure

## Discussion

The main findings in the present study are summarized as follows: PPV did not perform well enough to predict FR in the general population of patients who received controlled-volume mode with spontaneous efforts. Meanwhile, PPV accurately predicted FR in patients with low inspiratory efforts, especially in patients with P_0.1_ < 1.5 cmH_2_O.

The findings in our general population are in accordance with previous studies [21], although several differences exist. Unlike previous studies using the pressure support mode, all patients in the present study were ventilated using the volume-controlled mode, but they kept spontaneous breathing activity. The poor predictive performance of PPV in patients with spontaneous breathing is primarily attributed to the irregular changes of intrathoracic pressure, either in rate or in amplitude [5, 22], while controlled ventilation with spontaneous efforts can attenuate the irregularity as much as possible. Furthermore, such conditions are more compatible with real clinical situations, since patients are often unable to breathe under a pressure support mode at the early phase of their acute disease, when the question of fluid responsiveness is crucial to be answered. We also used strict exclusion criteria to exclude confounders (which can impact the performance of PPV) as much as possible, including cardiac arrhythmias, right heart dysfunction, and intra-abdominal hypertension. Besides, 75% of the included patients had a respiratory system compliance > 30 mL/cmH_2_O, which could explain the excellent performance of PPV in patients with low inspiratory effort.

The previous studies did not consider the impact of the magnitude of inspiratory effort when assessing the predictive performance of PPV. A marked inspiratory effort during mechanical ventilation can limit the use of PPV through numerous aspects. During the inspiratory phase, an enhanced inspiratory activity could increase the right ventricle (RV) preload and the ventricle (LV) afterload because of decreased intrathoracic pressure, which is opposite to the effect of mechanical ventilation without spontaneous breathing activity. Thus, in case of marked inspiratory effort the ability of PPV to predict FR could not be as good as in the case of fully controlled mechanical ventilation. Besides, an active expiratory contraction of abdominal muscles could drive blood from the abdominal compartment into the thorax and subsequently increase the RV preload and after a phase lag. All these factors may result in both false negative and false positive of PPV performance.

In the present study, we used P_0.1_ and ΔP_occ_ to reflect the magnitude of the inspiratory effort. The P_0.1_ easily obtained from the ventilator after expiratory occlusion is qualified to detect potentially excessive and low inspiratory effort in patients who undergo mechanical ventilation. Recent reviews defined weak spontaneous effort as P_0.1_ less than or equal to 1–1.5 cmH_2_O, and vigorous spontaneous effort as P_0.1_ great than or equal to 3.5–5 cmH_2_O [23, 24]. In the present study, we defined low effort as P_0.1_ less than 1.5 cmH_2_O (the median value of P_0.1_ in our cohort), which was very close to the previous threshold. The ΔP_occ_ is correlated with the pressure generated by the respiratory muscles to expand the lungs and chest wall, and the measurements are not affected by the type of ventilator [14]. ΔP_occ_ has been recently shown to accurately detect excessive respiratory muscle pressure, and the suggested target for lung and diaphragm-protective ventilation was − 20 to – 8 cmH_2_O for ΔP_occ_ [25]. Inconsistent with the result of P_0.1_, patients with ΔP_occ_ ≥ − 9.8 cmH_2_O did not exhibit a significantly higher performance of PPV compared to patients with ΔP_occ_ < − 9.8 cmH_2_O. All included patients were sedated and ventilated using the volume-controlled mode, and the range of ΔP_occ_ values might not be broad enough to detect a meaningful threshold.

The major strength of our study is the demonstration that PPV can still be reliable in mechanically ventilated patients with persistent low breathing activity. These results are valuable since persistent spontaneous breathing activity during mechanical ventilation is common, and PPV is easily obtained from conventional hemodynamic monitors in patients with an arterial catheter in place. Our results contradict the general principle that PPV is invalid in patients with spontaneous effort.

Our study has some limitations. First, our single-center study included patients that were all sedated and ventilated using the volume-controlled model, and a large proportion of patients did not exhibit strong inspiratory effort. Besides, the values of P_0.1_ measured by Servo-I might be different from the ventilators that perform a true occlusion to measure P_0.1_. The cut-off value of P_0.1_ (1.5 cmH_2_O) in our study may not always be applicable to other patients. Further studies are required to examine the generalizability of our findings. Second, the number of fluid responders was relatively low in our study compared to other previous studies. Non-responders experienced an unnecessary adrenergic burden at baseline in our study, which could impact the cardiac response to fluid challenges [26]. Besides, the fluid challenge consisted of a lower volume of fluids in the present study compared to previous studies [4], which could also decrease the number of fluid responders [27]. Third, we did not measure the intrathoracic pressure with an esophageal balloon. However, the association between P_0.1_ or ΔP_occ_ and intrathoracic pressure was demonstrated in previous studies [14, 28], and the non-invasive method we chose is more feasible in clinical practice. Finally, we assessed the inspiratory effort in patients who underwent controlled ventilation and received sedation, the range of P_0.1_ and ΔP_occ_ values was not broad enough to explore the impact of inspiratory effort on PPV performance, which needs further research.

## Conclusions

Our study shows that PPV did not perform well enough to predict FR in the general population of patients who received controlled ventilation with spontaneous effort. However, PPV was reliable in predicting FR in patients exhibiting a low inspiratory effort, especially in patients with a low value of P_0.1_, a parameter easy to be obtained at the bedside.

### Supplementary Information

**Additional file 1: Table S1.** Baseline Characteristics of included patients at enrollment stratified by fluid responsiveness. **Table S2.** Effects of volume expansion on hemodynamic parameters in fluid Responders and Non-responders. **Table S3.** Impact of different degrees of inspiratory effort on the correct classification of fluid responsiveness using the univariable logistical regression model. **Figure S1.** Patients selection in the study. **Figure S2.** Gray zone (8.5–15.5%) of pulse pressure variation (PPV) to predict fluid responsiveness in all patients.

## Data Availability

Data are available upon reasonable request and with the approval from the Department of Critical Care Medicine, Zhongda Hospital, School of Medicine, Southeast University.

## Abbreviations

PPV: Pulse pressure variation
FR: Fluid responsiveness
P_0.1_: Airway occlusion pressure
AUROC: Area under receiver operating characteristic
CO: Cardiac output
V_T_: Tidal volume
ICU: Intensive care unit
SBP: Systolic blood pressure
MAP: Mean arterial pressure
PBW: Predicted body weight
PEEP: Positive end-expiratory pressure
P_aw_: Airway pressure
ΔP_occ_: Occlusion pressure
NEE: Norepinephrine equivalent
HR: Heart rate
CVP: Central venous pressure
SOFA: Sequential organ failure assessment
