# Supplementary material for: Inspiratory effort impacts the accuracy of pulse pressure variations for fluid responsiveness prediction in mechanically ventilated patients with spontaneous breathing activity: a prospective cohort study
Source: Ann Intensive Care. 2023 Aug 17;13:72. doi: 10.1186/s13613-023-01167-0 (PMC10435426; doi:10.1186/s13613-023-01167-0)

Table S1: Baseline Characteristics ﻿of included patients at enrolment stratified by fluid responsiveness.

|  | Total patients  (n=189) | Responders  (n=53) | Non-responders  (n=136) | P value |
| --- | --- | --- | --- | --- |
| Age, yr | 66.3 (13.3) | 65.0 (14.4) | 66.9 (12.9) | 0.40 |
| Gender, male (%) | 121 (64.0) | 41 (77.4) | 80 (58.8) | 0.027 |
| ﻿BMI, kg/m^2^ | 23.8 (3.9) | 23.9 (4.3) | 23.7 (3.7) | 0.76 |
| APACHE II | 24.5 (6.3) | 25.1 (6.2) | 24.3 (6.4) | 0.54 |
| SOFA score | 9.9 (3.5) | 10.1 (2.8) | 9.8 (3.7) | 0.50 |
| RASS | -2.4 (1.3) | -2.2 (1.1) | -2.4 (1.4) | 0.29 |
| CPOT | 0 (0) | 0 (0) | 0 (0) | 1.00 |
| **Comorbidities** | | | | |
| Hypertension, n (%) | 93 (49.2) | 25 (47.2) | 68 (50.0) | 0.85 |
| Diabetes, n (%) | 81 (42.9) | 17 (32.1) | 64 (47.1) | 0.088 |
| Coronary heart disease, n (%) | 37 (19.6) | 11 (20.8) | 26 (19.1) | 0.96 |
| Chronic heart failure, n (%) | 53 (28.0) | 17 (32.1) | 36 (26.5) | 0.55 |
| Chronic renal disease, n (%) | 25 (13.2) | 15 (28.3) | 10 (7.4) | <0.001 |
| Stroke, n (%) | 36 (19.0) | 4 (7.5) | 32 (23.5) | 0.021 |
| ﻿**Acute circulatory failure origin, n (%)** | | | | |
| Septic shock | 167 (88.4) | 45 (84.9) | 122 (89.7) | 0.50 |
| Cardiogenic shock | 4 (2.1) | 2 (3.8) | 2 (1.5) | 0.67 |
| Neurogenic shock | 12 (6.3) | 2 (3.8) | 10 (7.4) | 0.57 |
| Hypovolemic shock | 10 (5.3) | 2 (3.8) | 8 (5.9) | 0.83 |
| **Respiratory parameters at enrolment** | | | | |
| FiO_2_ | 0.49 (0.17) | 0.48 (0.17) | 0.49 (0.17) | 0.77 |
| Tidal volume, ml | 425 (92) | 435 (87) | 421 (93) | 0.32 |
| Tidal volume/PBW, ml/kg | 7.0 (1.0) | 7.0 (1.0) | 7.0 (1.0) | 0.98 |
| Respiratory Rate (Set), bpm | 15.2 (2.4) | 15.5 (2.3) | 15.1 (2.5) | 0.35 |
| Respiratory Rate (Observed), bpm | 19.4 (5.3) | 19.9 (4.2) | 19.2 (5.7) | 0.41 |
| PEEP, cmH_2_O | 5.1 (0.5) | 5 (0.0) | 5.1 (0.6) | 0.44 |
| Peak Pressure, cmH_2_O | 24.4 (6.2) | 25.1 (6.4) | 24.1 (6.2) | 0.42 |
| Plateau Pressure, cmH_2_O | 17.7 (4.7) | 19.2 (4.5) | 17.2 (4.7) | 0.012 |
| Driving Pressure, cmH_2_O | 11.8 (4.6) | 13.0 (4.4) | 11.3 (4.6) | 0.027 |
| Compliance, ml/cmH_2_O | 37.2 [29.3, 48.8] | 35.5 [27.5, 43.3] | 37.6 [30.4, 50.8] | 0.084 |
| P_0.1_, cmH_2_O | 1.5 [0.8, 2.8] | 1.8 [0.9, 3.0] | 1.5 [0.6, 2.8] | 0.15 |
| ΔP_occ_, cmH_2_O | -9.8 [-14.0, -3.7] | -10.7 [-14.6, -5.9] | -8.4 [-13.9, -3.2] | 0.036 |
| PaO_2_/FiO_2_, mmHg | 223 [173, 287] | 222 [160, 270] | 255 [199, 308] | 0.069 |
| pH | 7.38 (0.08) | 7.38 (0.05) | 7.38 (0.09) | 0.92 |
| PaCO_2_, mmHg | 39.1 (12.6) | 39.9 (9.1) | 38.8 (13.7) | 0.59 |
| SB, mmol/L | 22.9 (4.7) | 24.0 (4.6) | 22.5 (4.6) | 0.046 |
| **Hemodynamic parameters at enrolment** | | | | |
| Heart Rate, beats/min | 98.4 (20.4) | 99.4 (23.1) | 98.0 (19.4) | 0.67 |
| HR/RR | 5.02 (1.23) | 5.01 (1.26) | 5.02 (1.22) | 0.97 |
| Vasopressor dose, μg/kg/min NEE | 0.33 [0.15, 0.57] | 0.38 [0.14, 0.71] | 0.33 [0.16, 0.50] | 0.55 |
| Lactate, mmol/L | 3.4 (2.6) | 3.2 (2.6) | 3.5 (2.7) | 0.40 |

BMI: Body mass index; APACHE II: Acute physiology and chronic health score II; SOFA: Sequential organ failure assessment; RASS: Richmond Agitation-Sedation Scale; CPOT: Critical Care Pain Observation tool; PBW: Predicted body weight; PEEP: Positive ﻿ end-expiratory pressure; P_0.1_: Airway occlusion pressure; ΔP_occ_: Airway pressure swing during a whole breath occlusion; PaO_2_/FiO_2_: Arterial partial pressure of oxygen/fraction of inspired oxygen; PaCO_2_: Partial pressure of carbon dioxide; SB: Sodium bicarbonate; HR/RR: Heart Rate/Respiratory Rate (Observed); NEE: Norepinephrine equivalent.

Table S2: ﻿Effects of ﻿volume expansion on hemodynamic parameters in fluid Responders and Non-responders.

|  | Responders  (n=53) | | Non-responders  (n=136) | |
| --- | --- | --- | --- | --- |
|  | Baseline | After Fluids | Baseline | After Fluids |
| SBP, mmHg | 124.7 (17.4) | 132.4 (19.1) | 134.1 (16.8)^$^ | 137.6 (17.9) |
| DBP, mmHg | 57.7 (11.2) | 59.5 (12.6) | 63.7 (13.6)^$^ | 64.6 (13.4) |
| MAP, mmHg | 78.7 (12.4) | 84.0 (13.5)^*^ | 88.9 (13.6)^$^ | 91.1 (13.6)^&^ |
| CVP, mmHg | 8.1 (4.0) | 8.3 (4.4) | 9.56 (3.8)^$^ | 10.94 (4.5)^#&^ |
| PPV, % | 13.3 (6.2) | 11.2 (5.3) | 7.53 (3.9)^$^ | 7.56 (4.0)^&^ |
| SVV, % | 13.3 (7.3) | 11.6 (5.4) | 7.40 (4.9)^$^ | 7.30 (4.3)^&^ |
| SV, ml | 61.4 (17.2) | 74.6 (21.3)^*^ | 76.2 (19.0)^$^ | 76.4 (20.0) |
| SVI, ml/m^2^ | 35.2 (8.9) | 42.9 (11.3)^*^ | 44.3 (9.5)^$^ | 44.3 (10.1) |
| ﻿Cardiac output, L/min | 5.44 (1.29) | 6.78 (1.91)^*^ | 6.63 (1.77)^$^ | 6.69 (1.86) |
| Cardiac index, L/min/m^2^ | 3.11 (0.68) | 3.89 (0.99)^*^ | 3.86 (0.91)^$^ | 3.90 (0.96) |
| SVR, dyn.s.cm^-5^ | 1043 [888, 1303] | 901 [757, 1097]^*^ | 966 [786, 1163] | 921 [805, 1144] |
| SVRI, dyn.s.m^2^.cm^-5^ | 1761 [1572, 2359] | 1538 [1312, 1908]^*^ | 1673 [1339, 2073] | 1582 [1353, 2041] |
| ITBV, ml | 1576 [1371, 1836] | 1722 [1435, 2042]^*^ | 1696 [1347, 1928] | 1690 [1357, 1875] |
| ITBVI, ml/m^2^ | 893 [714, 1092] | 977 [839, 1139] | 976 [857, 1082]^$^ | 964 [867, 1085] |
| GEDV, ml | 1261 [1098, 1469] | 1385 [1148, 1634]^*^ | 1357 [1078, 1544] | 1352 [1086, 1501] |
| GEDVI, ml/m^2^ | 716 [573, 869] | 782 [671, 912]^*^ | 784 [686, 872] ^$^ | 771 [694, 868] |
| EVLW, ml | 497 [433, 658] | 527 [412, 699] | 515 [385, 719] | 477 [359, 672] |
| EVLWI, ml/m^2^ | 8.5 [6.5, 9.7] | 8.8 [6.1, 11.1] | 8.1 [6.7, 11.3] | 7.8 [6.5, 11.1] |

^*^p<0.05: After Fluids versus Baseline (Responders);

^#^p<0.05: After Fluids versus Baseline (Non-responders);

^$^p<0.05: Non-responders versus Responders (at baseline)

^&^p<0.05: Non-responders versus Responders (After Fluids)

SBP: Systolic blood pressure; DBP: Diastolic blood pressure; MAP: Mean arterial pressure; CVP: Central venous pressure; PPV: ﻿Pulse pressure variation; SVV: Stroke volume variation; SV: Stroke volume; SVI: Stroke volume index; SVR: Systemic vascular resistance; SVRI: Systemic vascular resistance index; ITBV: Intrathoracic blood volume; ITBVI: Intrathoracic blood volume index; GEDV: Global end-dilution volume; GEDVI: Global end-dilution volume index; EVLW: Extravascular lung water; EVLWI: Extravascular lung water index.

Table S3: Impact of different degrees of inspiratory effort on the correct classification of fluid responsiveness using the univariable logistical regression model.

|  | **OR** | **95% CI** | **P value** |
| --- | --- | --- | --- |
| **P_0.1_** | | | |
| P_0.1_ < 1.5 cmH_2_O | Reference | — | — |
| P_0.1_ ≥ 1.5 cmH_2_O | 0.32 | 0.16-0.63 | 0.001 |
| **ΔP_occ_** | | | |
| ΔP_occ_ ≥ -9.8 cmH_2_O | Reference | — | — |
| ΔP_occ_ <-9.8 cmH_2_O | 0.43 | 0.22-0.82 | 0.011 |

P_0.1_: Airway occlusion pressure; ΔP_occ_: Airway pressure swing during a whole breath occlusion; OR: odds ratio; CI: Confidence Interval.

Figure S1: Patients selection of the study.


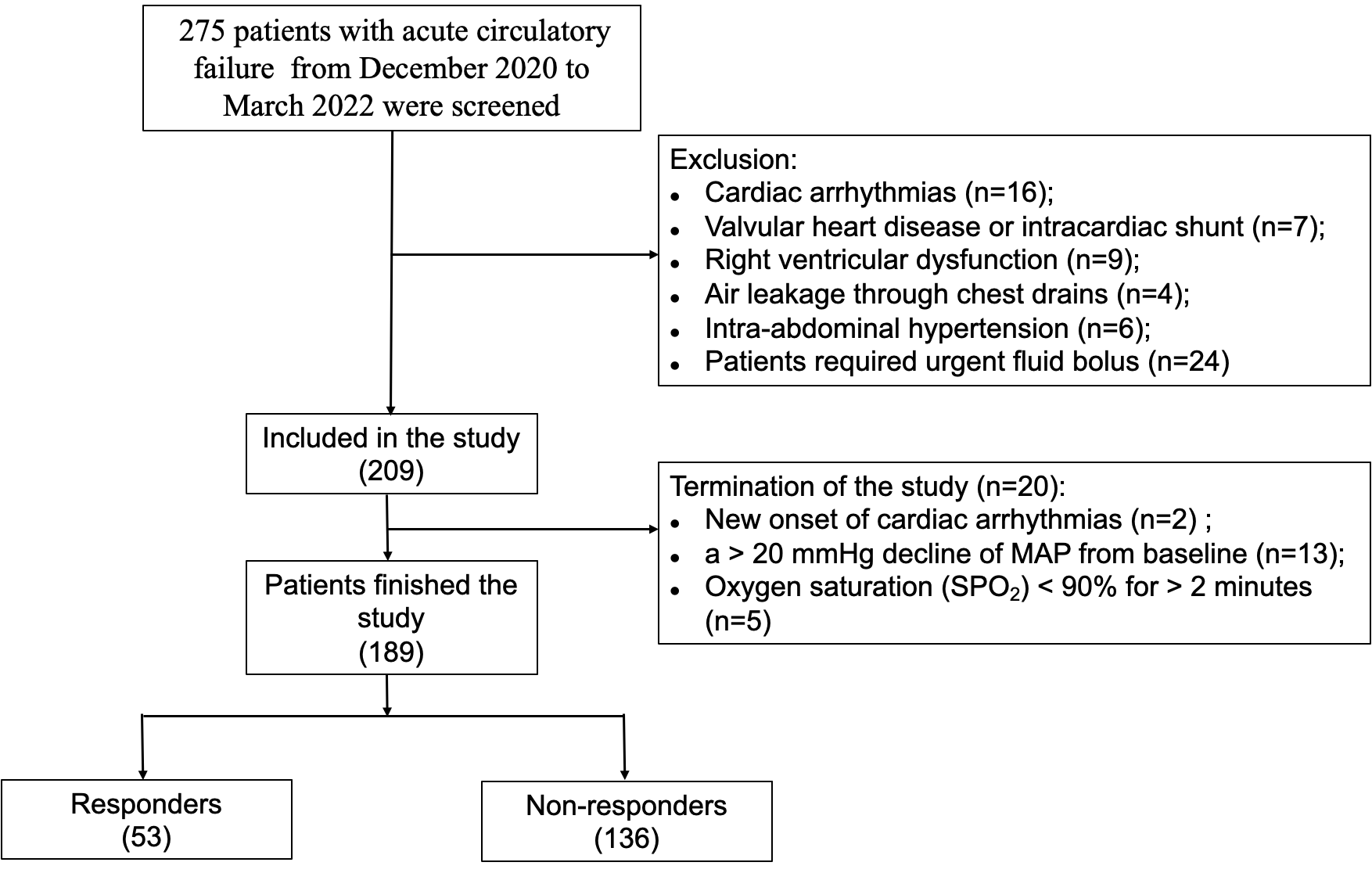


Figure S2: Gray zone (8.5-15.5%) of pulse pressure variations to predict ﻿fluid responsiveness in all patients


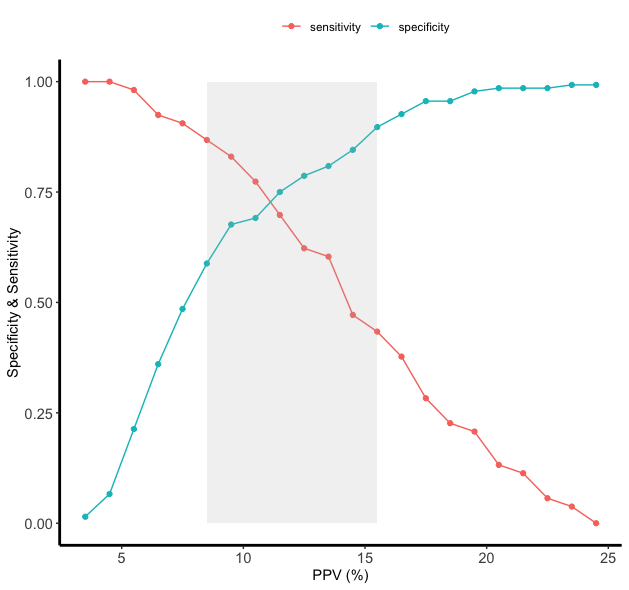

Supplement: Supplementary file 1 — Additional file 1: Table S1. Baseline Characteristics of included patients at enrollment stratified by fluid responsiveness. Table S2. Effects of volume expansion on hemodynamic parameters in fluid Responders and Non-responders. Table S3. Impact of different degrees of inspiratory effort on the correct classification of fluid responsiveness using the univariable logistical regression model. Figure S1. Patients selection in the study. Figure S2. Gray zone (8.5–15.5%) of pulse pressure variation (PPV) to predict fluid responsiveness in all patients. [file 13613_2023_1167_MOESM1_ESM.docx]
